# Characterization and Cytotoxicity Analysis of a Ciprofloxacin Loaded Chitosan/Bioglass Scaffold on Cultured Human Periodontal Ligament Stem Cells: a Preliminary Report

**DOI:** 10.3889/oamjms.2016.052

**Published:** 2016-05-22

**Authors:** Maha I. Abdelfattah, Sherine A. Nasry, Amani A. Mostafa

**Affiliations:** 1 *Oro-dental Genetics Department, Human Genetics and Genome Research Division, National Research Centre, Cairo, Egypt*; 2 *Surgery and Oral Medicine Department, Oro-dental Research Division, National Research Centre, Cairo, Egypt*; 3 *Refractories, Ceramic & Building Materials Department & Nanomedicine & Tissue Engineering Laboratory, MRCE, National Research Centre, Cairo, Egypt*

**Keywords:** stem cells, periodontal, cytotoxicity, ciprofloxacin, chitosan

## Abstract

**AIM:**

The aim of this study was to analyze the cytotoxicity of ciprofloxacin (CIP) loaded on chitosan bioactive glass scaffold on human periodontal ligament stem cells (PLSCs) in vitro.

**MATERIALS AND METHODS:**

PLSCs obtained from human third molars, cultures treated with medium containing 15 x 15 mm chitosan/bioactive glass scaffolds without/with different concentration 0, 5, 10, and 20 % of CIP. A total of 15 x 10^3 cells were plated in 6 well plates. The attached cells of each group were harvested from the plates after 1, 4 and 8 days of culture to detect the viability of cells. The cell number was determined using a hemocytometer and the trypan blue dye-exclusion assay. Data was analyzed using normality using Shapiro-Wilk test. Comparisons between groups were made using One-way ANOVA complemented by Tukey’s test.

**RESULTS:**

When comparing the proliferation rate of cells in the four groups, no statistically significant difference was found (P = 0.633). With regards to cell viability, no statistical difference was found between the 0, 5, and 10 % CIP concentrations, while the 20 % CIP concentration demonstrated the least viability with a high statistically significant difference (P = 0.003).

**CONCLUSION:**

Twenty percentages CIP demonstrated the least proliferation rate and viability.

## Introduction

Periodontitis is a complex disease which occurs when environmental and bacterial factors with allelic variants of multiple genes act synergistically to increase or decrease the likelihood of developing a disease [1] Dental pathogens are not planktonic but form complex communities called biofilms that cause chronic infections by resisting antibiotic treatments and killing by the host immune system [2].

Aggressive periodontitis (AgP) is a type of periodontal diseases that causes rapid destruction of the periodontal attachment apparatus and the supporting alveolar bone. The familial nature of AgP has led to speculation that a major gene defect is responsible for its transmission and many genetic disorders are associated with AgP such as Papillon Lefevre syndrome and Down syndrome [1]. Patients with AgP often present with limited microbial deposits that seem inconsistent with the severity of tissue destruction but often have elevated levels of Aggregatibacter actinomycetemcomitans (Aa), that possess variety of virulence factors that can impair PMNL’s function and potentiate the disease process [3] and does not respond to mechanical therapy alone as these pathogens have been found to be invasive and re-infect the pocket. Thus, augmenting mechanical therapy with antibiotics should be used to eliminate the pathogens left in the tissues [4].

Many clinical studies and systematic reviews have attested to the beneficial effects of using systemic as well as local antibiotics as an adjunctive therapy to mechanical scaling and root planning in terms of pocket reduction and attachment gain, and these effects are more pronounced in AgP patients. However, it has been reported that the benefits of systemic antibiotics administrated in periodontal therapy should be balanced against the possible side effects of the repeated use of these antibiotics in treating periodontal diseases, and that in comparison to systemic antibiotics, the application of antimicrobials by sustained-delivery devices may offer a better pocket depth reduction and gain in attachment level without the side effects seen in systemic antibiotics [5].

It has been found that both systemic antibiotics and topical chlorhexidine in patients with AgP did not reduce the percentage of invaded epithelial cells, which points out the fact that intracellular reservoirs of bacteria exist and may lead to disease recurrence and/or refractory treatment in these patients. This invasion might cause the bacteria to withstand systemic antibiotic treatment [6].

Porous three-dimensional scaffolds have the ability to attach to cells and allow their proliferation [7]. Chitosan (C), a deacetylation derivative of chitin has gained much attention as a functional material for biomedical applications due to its non-antigenicity, biocompatibility and the ability to support cell attachment and proliferation [7]. It is a potential candidate for targeting antibiotic resistant microorganism due to a broad spectrum of antimicrobial activity [8]. Bioactive glasses (G) are ideal candidates for regeneration and the incorporation of these glasses into a chitosan scaffold have been shown to provide a backbone for the scaffold [9]. Ciprofloxacin (CIP), a fluoroquinone is more effective against gram-negative bacteria, particularly Aa [10]. It is the only antibiotic used in periodontal therapy to which all strains of Aa are susceptible [11]. CIP level in the gingival crevicular fluid was also demonstrated to be significantly higher than in serum [12]. Antibiotics loaded on scaffolds and released into the periodontal pocket should not be toxic to the living cells [13]. Cells from the pulp and apical papilla were shown to be susceptible to CIP toxic effects. These effects also depend on the dosage and time of exposure of these cells to CIP [12, 13].

The purpose of this study was to evaluate the cytotoxic effect of different concentration of CIP loaded on a chitosan/glass scaffold on the proliferation and viability of PLSCs.

## Materials and Methods

### Fabrication of scaffold

Tetraethyl orthosilicate (TEOS: Fluka, wt = 208.33), calcium nitrate hydrate (Fluka, M.wt = 236), sodium hydroxide (Prolab, M.wt = 40) and ammonium dihydrogenphosphate (MERK, M. wt = 115.03) in addition with chitosan of medium molecular weight were used in this study. The 46S6 bioactive glass with the composition (46% SiO_2_, 24% CaO, 24% Na_2_O, 6% P_2_O_5_wt%) was prepared by sol-gel technique and named (S) [16]. A mixture of chitosan (C) in a concentration of 3% w/vand the previously prepared bioactive glass (G) of the ratio 1:2 was used in this study. CIP in different ratios: 0, 5, 10 and 20 wt% were added to the above formula during preparation and named; CIP- 0%, CIP -5%, CIP -10% and CIP -20%, respectively. The freeze drying technique was used to get porous scaffolds. The composition of all the prepared scaffold formulations was cast in a mould with dimensions1.2 x 0.3 cm, kept at -80°C for overnight, and freeze dried at the same temperature for 24 h before further cell culture analysis [15,16].

### Characterization

#### Scaffold morphology by SEM

Scanning electron microscope (SEM) coupled with energy dispersive spectroscopy (EDS) was used for morphological evaluation and elemental analysis. SEM analyses were performed on the structure of the drug and the surface of scaffolds without/with CIP.

#### Ciprofloxacin releases from the investigated scaffolds

Phosphate buffer saline (PBS) with pH 7.4 at 37°C was used to investigate the CIP release from the scaffolds. The scaffolds were immersed in 10 ml PBS and shacked by an incubator shaker at 100 struck per minute. Samples were removed at different time intervals and the drug concentration was determined using spectrophotometer at 277 nm [19].

### Sampling and Cell Culture

Healthy two lower right impacted molars, (as it is the latest erupted teeth and has the youngest cells), were extracted a traumatically, (to avoid loss of periodontal ligament during the extraction procedure), from two different healthy female patients, aged from 18-25 years. The teeth were extracted for patients attending the Clinic of the Oral and Maxillofacial Surgery Department, National Research Centre, Egypt and informed consent was obtained from the patients before extraction. The extracted teeth were immediately put in a sterile 50 ml polypropylene tube supplemented with culture media (DMEM) (Lonza Bioproducts, Belgium). Then, the teeth were transported to the cell culture laboratory in about 20 minutes.

With totally aseptic conditions, the periodontal ligament tissue was minced into pieces and digested in a solution of 2 mg/ml Collagenase NB 4 (SERVA, crescent chemical company, USA) for 30 minutes at 37°C in a water bath. Single cell suspension was seeded into a T-25 flask (Costar, Cambridge, USA) with DMEM supplemented with 10% fetal bovine serum (FBS) (Equitech-Bio Inc., Kerrville, USA), 100 U/ml penicillin, 100 µg/ml streptomycin and 1% Fungizone (Lonza Bioproducts, Belgium), then incubated in 5% carbon dioxide incubator at 37°C. The samples were sub-cultured after reaching 80-85% confluence by utilizing 0.25% trypsin and 0.02% EDTA to get the next passage of cells. The third and fourth passages were used in all the coming procedures.

### Detection of the self-renewal capability of PLSCs

For this assay, 100,000 cells counted by hemocytometer were plated on a T-25 flask and incubated for 10 days. Subsequently, the cultures were fixed with 4% paraformaldehyde and afterward stained with 0.1% toluidine blue. Aggregates superior to or equivalent to 50 cells were scored as colonies [20].

### Identification of PLSCs by immunocytofluorescence

PLSCs were sub-cultured into 24- well cell culture plates, just as cells attained semi-confluency they were fixed with 4% paraformaldehyde for 30 min, afterward blocked with PBS containing 10% goat serum at room temperature for 30 min, and then incubated with primary antibody (Anti-STRO-1, mouse monoclonal IgM anti-human STRO- 1) (Millipore, Darmstadt, Germany) at dilution 1 μl: 200 μl of PBS for overnight at 4°C. Following washing, the samples were incubated with fluorescein- conjugated secondary antibody (goat anti-mouse IgM) for 60 minutes in the dark. Regarding nuclei staining, DAPI (4, 6-diamino-2-phenylindol) was used for five minutes. Following washing with PBS, the samples were examined by fluorescence microscopy with 40 X original magnification [21].

### Multilineage differentiation

For osteogenic differentiation in vitro, PLSCs cultures were supplemented with 0.01 µmol/L dexamethasone, and 1.8 mmol/L inorganic phosphates (Sigma-Aldrich, United States). The medium was changed twice weekly. After that, the samples were stained after 14 days by 2% alizarin red stain to detect calcium accumulation in vitro [22]. For adipogenic differentiation in vitro, PLSCs cultures were supplemented with 0·5 μmol/L isobutylmethylxanthine, 0·5 μmol/L hydrocortisone, 60 μmol/L indomethacin and 10 μg ml insulin (Sigma-Aldrich, United States) for 21 days to induce adipogenic differentiation, then Oil red O staining was applied to identify lipid-laden fat cells [21].

### Cytotoxic analysis of scaffold-CIP on cultured human PLSCs.

The scaffolds (15x15 mm) were first sterilized by UV radiation of the laminar flow (30 min for each side), followed by 30 min immersion in 70 % ethanol [23]. A total of 15 x 10^3^ cells were plated in 6 well plates, after 24 hours of culture, the culture medium was changed. Afterward, chitosan bioglass loaded with different ciprofloxacin concentrations (5, 10, and 20%) and 0 % (positive control) was added. After 1, 4 and 8 days of culture, the cells were harvested and the cell number was determined by counting the viable cells by a hemocytometer using the trypan blue dye-exclusion assay, where 100 micro liter of trypan blue was mixed to 100 micro liter of treated cellular suspension and left for 10 minutes, then 10 μl of the mixture was spread into both chambers of the hemocytometer. Subsequently, the hemacytometer was observed under an inverted light microscope using the 20 x objective lens. The number of viable cells harvested was obtained by the following equation: UC×D×104/nSQ, where UC is the unstained cell count (viable cells), D is the dilution of the cell suspension, and nSQ is the number of counted squares in the hemocytometer. Viable cells appear colorless and bright under phase contrast microscopy. Nonviable cells appear blue-stained and are non-refractile. The viable percentage of the cell population was obtained using the equation: UC/TC× 100, where UC is the unstained cell count (viable cells) and TC is the total cell count (stained plus unstained cells) [24].

### Statistical Analysis

Analysis of data was performed utilizing SPSS 18 (Statistical Package for Scientific Studies) for Windows. Data were explored for normality using Shapiro-Wilk test. Comparisons between groups were made utilizing One-way ANOVA complemented by Tukey’s test. The level of significance was p < 0.05.

## Results

**Figure 1:**
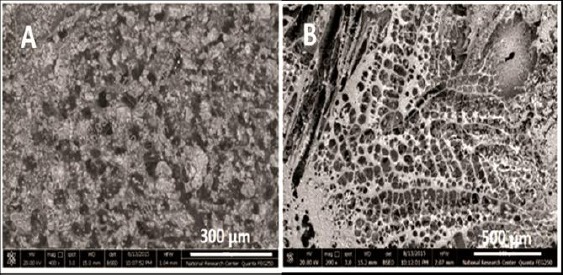
Scanning Electron Micrograph of scaffold without ciprofloxacin (CIP-0%), the glass particles homogeneously distributed as in (A) and the porosity are in the range of 40-60 µm (B), magnification 300 µm and 500 µm, respectively

Representative SEM micrographs of the synthesized scaffolds (CIP-0%) are presented in (Fig. 1), where it can be noticed that the addition of (G) to the (C) scaffold gave a homogeneous structure of the scaffold with a rough texture compared to the smooth structure of a previously prepared chitosan scaffold alone. The mean pore diameter of the prepared scaffolds ranged between 40-60 µm, pointing to the wide range of interconnected pores of the scaffolds which can facilitate cell migration, adhesion, and proliferation.

**Figure 2:**
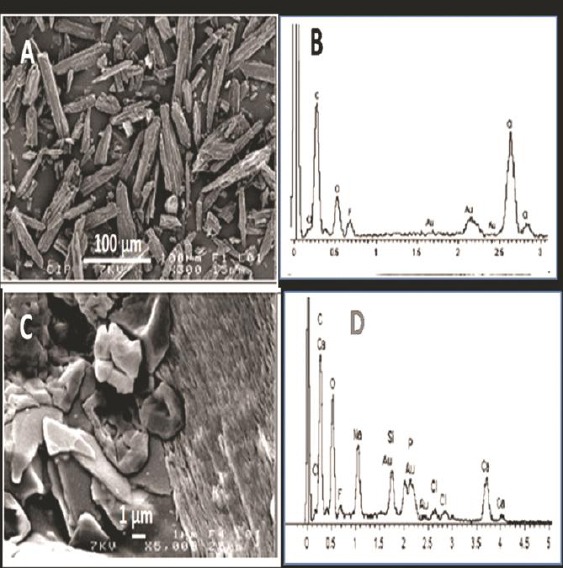
SEM micrographs and EDS of: the drug ciprofloxacin, CIP (A, B) with magnification 100µm, rod shape of the drug (A) elements of C and O appears in (B) and for chitosan/ bioactive glass scaffold loaded with CIP-20% (C,D) with magnification 1 µm, the glass crystals appear and the rod shape of the drug embedded in the chitosan matrix (C) and the elements of Ca, Na, Si and P of the glass beside that of the drug C and O (D)

The SEM micrographs and the elemental analyses of the CIP drug denoted in Fig. 2A,B, while the prepared scaffold loaded with (CIP-20%) presented in Fig. 2C, D. The rod shape of the drug could be seen, where the elemental analysis proved the presence of carbon (C) and oxygen (O) of the drug.

However, addition of the glass in the scaffold was confirmed by the presence of silicon (Si), phosphorous (P), sodium (Na) and calcium (Ca) in addition to (C) and (O) of the drug, which indicates the homogenous incorporation of the glass in the structure of the scaffold. CIP concentrations above 5 wt% led to the formation of fibers with significantly smaller diameters than PDS (p < 0.001).

In our assay, it is obvious that the drug release increased with increasing the drug concentration (from 5, 10 and 20 %). This is attributed to the difference between the concentration of the drug in the scaffold and that in the surrounding media, which could be explained by the large driving force of the drug release.

### Cell culture

PLSCs were successfully isolated from extracted impacted teeth. By observation of PLSCs under inverted microscopy, they appeared as fibroblast-like shaped cells after 3 days of culturing, which are characteristic to PLSCs. The cultured cells were rapidly proliferated till became confluent about 80%-90% after 7 days of culturing.

### Colony forming unit-fibroblast (CFU-F) assay

**Figure 3:**
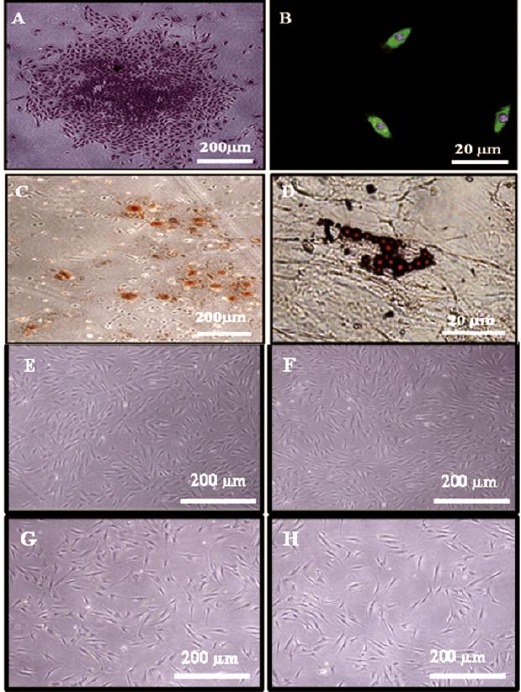
(A) Phase contrast micrograph showing a colony formed by PDLSCs after staining by toluidine blue stain. (4X). (B) Phase contrast micrograph showing PDLSCs express the MSC marker STRO-1 (Green colour). Notice counter stain of the nuclei by DAPI (Blue colour). (Indirect immunocytofluorescence, 40X). (C) Phase contrast micrograph showing extracellular calcium accumulation (Orange - red nodules) by differentiated PLSCs after 14 days of osteogenic induction (Alizarin red stain, 4X), and (D) Fat droplets formed by PLSCs after 21 days of adipogenic induction (oil red O stain, 40X). Phase contrast micrograph showing PLSCs after 8 days of culturing with scaffold loaded with (E) 0% Cipro, (F) 5% Cipro, (G) 10% Cipro and (H) 20% Cipro, maintained their PLSCs morphological characteristics

PDLSCs possess the potential to form clonogenic cell clusters, detected by the development of about 120 colonies, engendered from 100,000 single cells after culturing at low density, suggesting the large proliferative capacity for PLSCs. The cells within each colony were defined with a fibroblast-like morphology as revealed in (Fig. 3A).

### Immunocytofluorescence

Indirect immunocytofluorescence technique was utilized to identify STRO 1 antigen. In the current research, PDLSCs that was isolated were discovered to express the mesenchymal stem-cell marker STRO -1 as demonstrated in (Fig. 3B).

### Multilineage differentiation

On behalf of the osteogenic potential of PLSCs, our findings revealed that isolated PDLSCs revealed positive staining for calcium accumulation in vitro by utilizing Alizarin red stain after 14 days of culturing in osteogenic media (Fig. 3C). In addition, for the adipogenic potential of PLSCs, our results presented that PLSCs expressed oil red O-positive lipid clusters after 21 days of induction (Fig. 3D).

### Cytotoxic analysis of scaffold-CIP on cultured human PLSCs

Results are summarized in Tables 1 & 2. PLSCs after 8 days of culturing with CIP with different concentrations retained their PLSCs morphological characteristics compared to control untreated cells as shown in Fig. 3 E,F,G,H. After 24-hour time period, CIP 0%, CIP 5%, CIP 10% resulted in cell proliferation rates of 85×10^3^, 65×10^3^, 55×10^3^, respectively and viabilities of 100 % in all three groups, while the CIP group of 20 % concentration resulted in cell proliferation rate of 50 ×10^3^ and viability of 98 %. After 4 days, the cell proliferation rates of the CIP 0%, CIP 5%, CIP 10% and CIP 20% ciprofloxacin groups resulted in 180×10^3^, 120×10^3^, 90×10^3^ and 80 ×10^3^ respectively, while the cell viabilities were 100% in the CIP 0% and CIP 5% concentration and 99 and 98 % in the CIP 10 % and CIP 20 % concentration groups, respectively. At the 8^th^ day, the CIP 0%, CIP 5%, CIP 10% and CIP %20 concentration groups showed variable cell proliferation rates of 295×10^3^, 245×10^3^, 180×10^3^, and 165×10^3^, respectively. The cell viability remained 100% in the CIP 0% and CIP 5% concentration, and also remained at 99 % for the third group CIP 10% at 99%, while it showed the least viability in the CIP 20 % group at 96%.

**Table 1:** The cell proliferation rate for tested scaffolds at different time intervals

Group	Day 1	Day 4	Day 8	P-value
CIP- 0%	85 x 10^3^	180 x 10^3^	295 x 10^3^	0.633

CIP- 5%	65 x 10^3^	120 x 10^3^	245x 10^3^	

CIP- 10%	55 x 10^3^	90 x 10^3^	180 x 10^3^	

CIP- 20%	50 x 10^3^	80 x 10^3^	165x 10^3^	

**Table 2:** Cell viability for tested scaffolds at different time intervals

		Cell viability at:		

Group	Day 1	Day 4	Day 8	P-value
CIP- 0%^a^	100%	100%	100%	0.633

CIP- 5%^a^	100%	100%	100%	

CIP- 10%^a^	100%	99%	99%	

CIP- 20%^a^	98%	98%	96%	

The same superscript letters indicate statistically non-significant values (p > 0.05).

When comparing the proliferation rate of cells between the four concentrations groups, no statistical significance difference was found (P = 0.633). With regards to cell viability, no statistical difference was found between the 0, 5, and 10 % CIP concentrations, while the 20 % CIP concentration demonstrated the least viability with a high statistical difference (P = 0.003).

## Discussion

Aggressive periodontal diseases are caused by particular groups of microorganisms as A.a which is not eliminated by mechanical means alone due to the bacterial invasion, accordingly, adjunct anti-infective therapy is recommended. Periodontal disease treatment necessitates an anti-infective agent to infection sites and sustaining its localized concentration at effective levels for a sufficient time whereas at the same time evoking minimal or no side effects [25].

Unfortunately, the regular systemic antibiotic protocol for the treatment of AgP was found to be not effective in eradicating bacteria that reside within the epithelial cells. Although such treatment resulted in a reduction of bacteria and improved the clinical parameters, the number of invaded epithelial cells was not reduced [6]. Thus, local antibiotics are proposed compared to systemic antibiotics in terms of providing adequate dose and time to eradicate the infection and, also, prevent relapse without the side effects [26].

The use of drug delivery systems containing antimicrobial agents is advocated as it aims for releasing a sufficient level of the drug directly inside the periodontal pocket. Moreover, increasing the exposure of target microorganisms to higher concentrations could be realizing, thus minimizing the side effects associated with systemic drug administration. This, also, helps in patients where there is an intolerance to systemic administration and serves as a treatment option for localized areas of exudation and deep pockets not responding well to mechanical and systemic antibiotics [23, 24].

The use of antibiotic-loaded scaffold combined with bioactive glass as targeting drug-delivery systems for treatment of AP may allow the release of the drug directly into the periodontal pocket thus minimizes undesirable side effects caused by systemic drug administration and may also enhance patient compliance. However, these drugs should not exert any toxic effect on the underlying tissue. Therefore, this study aimed at evaluating the cytotoxic effects of different concentration of CIP on the viability and rate of proliferation of PDL stem cells. CIP was selected in the present study because, at present, it is the only antibiotic in periodontal therapy to which all strains of A.a. are susceptible. Previous results suggested that exposure of periodontal surfaces to CIP reduced the micro-colony size and cell surface density of A.a in the biofilm. Moreover, A.a resistance to CIP is rare or non-existent. It was also suggested that CIP retained bactericidal activity inside PMNs ultimately contributed to the enhanced intracellular killing of susceptible bacteria [10].

The appropriate scaffold fabrication technique is a critical choice as it can significantly influence the scaffold properties and its degradation rate [29]. In a former study fibroblasts attached successfully to chitosan proving that it is not only a biocompatible but also a successful tissue engineering scaffold [30]. In the present study, a chitosan/glass scaffold loaded with different concentration of CIP was formulated using the freeze-drying technique. This technique provides a convenient and easy mean for formulating highly porous scaffold, with interconnected pores, that has the ability to enhance regeneration. The bioactive glass was incorporated into the chitosan scaffold to adjust the quality of pores, the mechanical strength and degradation rate of chitosan scaffolds through [31].

In vitro testing using primary cells, as stem cells, to test the cytotoxicity of dental materials is more preferable than using established cell lines as these cells have been cultured for the first time, and are therefore similar to their original tissue. They are characterized by their largely unchanged metabolic status, and a high degree of differentiation [24]. PLSCs were used as target cells in the present study because they are easily accessible and are able to differentiate into other types of dental cells [32]. In the present study, CFU-F assay realized the successful self-renewal capacity of PLSCs from the primary cell culture, as well as indirect immunocytofluorescence, revealed that PLSCs are stained positive for the early MSC marker (STRO-1). Additionally, multilineage differentiation (osteogenic and adipogenic) of PLSCs was emphasized. The osteogenic potentiality was detected by orange – red nodules of calcium accumulation in vitro using alizarin red satin after 14 days of induction. The adipogenic potentiality of PLSCs was shown through their positive lipid clusters after 21 days of induction. These findings demonstrated that isolated periodontal ligament cells fulfill the criteria of mesenchymal stem cells. Our results are in agreement with other investigators [17, 18]. The trypan blue exclusion staining technique was chosen to differentiate nonviable from viable cells as it is a preferred assay due to its quickness and ease of performance [24].

It was found that the process of drug release increased when the concentration of CIP increased from 5 to 20 % which was related to the increase in the concentration gradient between the drug in the scaffold and the surrounding medium. Dental cells were found to be susceptible to the toxic effects of CIP in terms of time and dosage, where decreasing drug concentrations and time of exposure may significantly improve stem cell viability [14]. In the present study high concentration of CIP (20%) decreased growth rate and viability of PLSCs. In a former study, 10 and 20 mg/ml concentration of CIP significantly reduced the rate of growth of human embryonic stem cells but did not affect their viability and differentiation characteristics. The cytotoxic effect of CIP was however reversed when the antibiotic was withdrawn and the cells regained their normal growth rate [13]. Also, previous data demonstrated that high concentrations of antibiotics are harmful to the survival of stem cells of the apical papilla and that it is important that when using bactericidal medications they should not have a detrimental effect on the stem cell viability [15].

In conclusion, based on the results of this study we suggest that 5% CIP concentration might be a suitable concentration because it was less toxic to the cells than other concentrations. Our results, however, are preliminary and require further preclinical and in vivo research, to confirm the safety and effectiveness of this drug and ensure that its biological activity is retained in the delivery system.
